# Lumbar spondylolisthesis: STATE of the art on assessment and conservative treatment

**DOI:** 10.1186/s40945-021-00113-2

**Published:** 2021-08-09

**Authors:** Carla Vanti, Silvano Ferrari, Andrew A. Guccione, Paolo Pillastrini

**Affiliations:** 1 Department of Biomedical and Neuromotor Sciences (DIBINEM), Alma Mater Studiorum, University of Bologna, 40138 Bologna, Italy; 2 Department of Rehabilitation Science, College of Health and Human Services, George Mason University, Fairfax, VA 22030 USA

## Abstract

**Introduction:**

There is weak relationship between the presence of lumbar spondylolisthesis [SPL] and low back pain that is not always associated with instability, either at the involved lumbar segment or at different spinal levels. Therefore patients with lumbar symptomatic SPL can be divided into stable and unstable, based on the level of mobility during flexion and extension movements as general classifications for diagnostic and therapeutic purposes. Different opinions persist about best treatment (conservative vs. surgical) and among conservative treatments, on the type, dosage, and progression of physical therapy procedures.

**Purpose and importance to practice:**

The aim of this Masterclass is to provide clinicians evidence-based indications for assessment and conservative treatment of SPL, taking into consideration some subgroups related to specific clinical presentations.

**Clinical implications:**

This Masterclass addresses the different phases of the assessment of a patient with SPL, including history, imaging, physical exam, and questionnaires on disability and cognitive-behavioral components. Regarding conservative treatment, self- management approaches and graded supervised training, including therapeutic relationships, information and education, are explained. Primary therapeutic procedures for pain control, recovery of the function and the mobility through therapeutic exercise, passive mobilization and antalgic techniques are suggested. Moreover, some guidance is provided on conservative treatment in specific clinical presentations (lumbar SPL with radiating pain and/or lumbar stenosis, SPL complicated by other factors, and SPL in adolescents) and the number/duration of sessions.

**Future research priorities:**

Some steps to improve the diagnostic-therapeutic approach in SPL are to identify the best cluster of clinical tests, define different lumbar SPL subgroups, and investigate the effects of treatments based on that classification, similarly to the approach already proposed for non-specific LBP.

## Introduction

Spondylolisthesis (SPL) is the term employed to define a displacement of the vertebral body in reference to the bordering vertebral bodies. Meyerding classified SPL in relation to the amount of vertebral slippage related to the caudal vertebrae measured by plain radiography. Grade I corresponds to less than 25%, grade II to 25–50%, grade III to 51–75%, and grade IV to 76–100% slippage [1].

SPL is defined isthmic or degenerative, based on its aetiology. Isthmic SPL is the consequence of a spondylolysis, which is a congenital defect or post-traumatic break in the pars interarticularis. Spondylolysis is the most common “specific” pathology within the adolescent population complaining of low back pain (LBP) [2, 3]. Frequency of spondylolysis is higher among athletes who perform movements involving repeated spinal flexion and extension [4, 5].

Degenerative SPL is mostly caused by degenerative arthritis or disorders of the disc space. In adulthood and elderly, SPL is associated with degeneration of facet joints, smaller stabilizer muscle thickness at rest and during contraction, and overuse of stabilization muscles [6–8]. Multifidus atrophy has been reported in several studies on patients with SPL [8–10], and a reduction of the force of global back muscles may lead to, or aggravate, forward slipping in isthmic and degenerative SPL [9–12].

The increased mobility of the slipped vertebra and the antero-inferior pressure on the disc may provoke increased pressure on the spinal nerve and reduction of intervertebral foramina. Patients with isthmic and degenerative SPL can develop both radicular symptoms due to the compression of the nerve root and neurogenic claudication due to lumbar spinal stenosis, caused by the slippage, the hypertrophy of the ligamentum flavum, and/or osteophytes [13], although these symptoms are not related to the amount of slippage [14].

SPL may be or not associated with spinal pain, and therefore is defined symptomatic or asymptomatic respectively. The natural history of SPL is generally favorable and only 10–15% of patients seeking treatment will have surgery [15]. The percentage of incidence rate of progression was reported as 34% in degenerative SPL, 32% in congenital isthmic SPL, and 4% in post-traumatic isthmic SPL [16].

There are still different opinions about best treatment options (conservative vs. surgical); and among conservative treatments, on the type, dosage, and progression of physical therapy procedures. Despite the ongoing debate on the definition and treatment of lumbar instability, the literature commonly correlates the symptoms provoked by lumbar SPL to reduced lumbar stability.

**Fig. 1:**
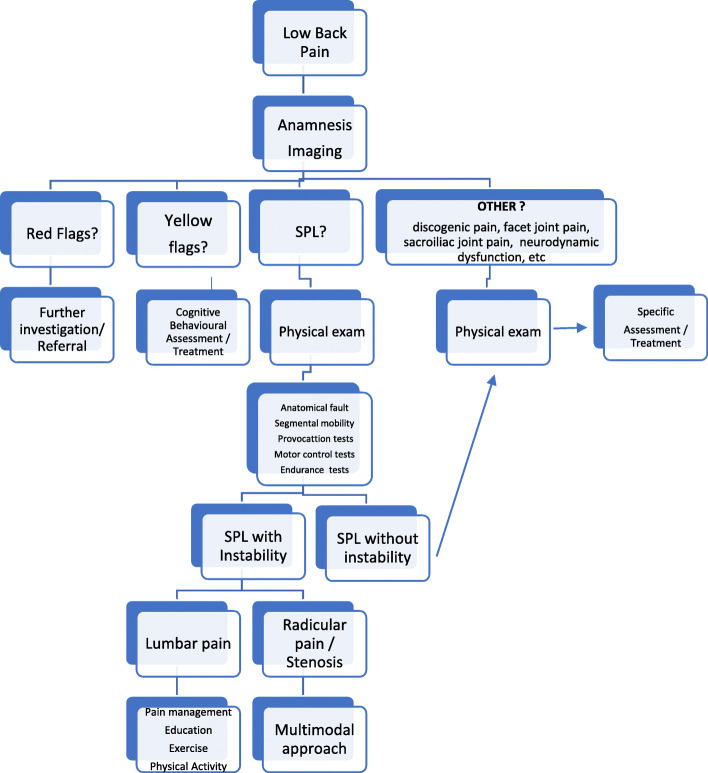
Algorithm showing the diagnostic/therapeutic process

Frequently, hypermobility at the SPL level is compensated by hypomobility of other spinal levels, mostly the thoracic ones, and vice-versa [17]. Hypermobility of the segments adjacent to the one involved by SPL also has been observed [18]; even so, SPL is not always associated with instability, both at the involved lumbar segment and at different spinal levels. Phan and colleagues divided SPL patients into stable and unstable groups, based on the level of mobility during flexion and extension movements [18]. This can be assumed as a general classification for an algorithm relevant to diagnostic and therapeutic processes (see Fig. 1).

SPL is common in neurosurgical, orthopedic and physical therapy and rehabilitation clinics; assessment and conservative intervention of patients diagnosed with SPL are usually standardized in clinical practice, despite different clinical characteristics. Classification of patients complaining of LBP into clinical subgroups based on signs and symptoms is considered important and current guidelines suggest tailored treatments for each specific condition according to individual clinical findings [19]. The aim of this Masterclass is to provide evidence-based indications for assessment and conservative treatment of SPL to clinicians, taking into consideration some subgroups related to variations in clinical presentations.

## Assessment

Assessment of a patient with symptomatic lumbar SPL includes history, imaging, and physical exam, which should also help to identify the so-called red and yellow flags. Red flags are signs and symptoms that may raise suspicion of serious spinal pathology (e.g. cauda equina syndrome, fracture, malignancy, and infection) and indicate that further investigation or referral is warranted. A recent framework by Finucane and colleagues suggests the most relevant findings related to low or high clinical suspicion for red flags in spinal pathologies [20].

Yellow flags indicate psycho-social obstacles to recovery and can be related to passive coping strategies, pain catastrophizing, fear-avoidance believes, poor self-efficacy, anxiety, and depression as well as environmental factors (related to family and work). Self-efficacy and active coping are protective factors for quality of life in chronic LBP patients [21, 22], while fear-avoidance beliefs and passive coping are considered risk factors [21]. Patients with chronic LBP show poor self-efficacy and heightened fear of movement [23, 24] and these issues may be present also in SPL due to an awareness of vertebral slipping and fear of damage [25].

Pain location alone does not help in differentiating symptomatic lumbar SPL from non-specific LBP. In fact, pain may be located both in lumbar area and/or referred to the lower limb/s. Taking into consideration that LBP comes from different causes, other characteristics must be considered to do a differential diagnosis between conditions similar to non-specific LBP (in which SPL is present but not relevant for the symptoms’ characteristics), and other conditions in which LBP is logically related to SPL, when lumbar instability and its consequences are the most important findings. Concerning the first condition, a clinician could expect a worsening of symptoms in discogenic pain by forward bending, whereas pain due to facet joints degeneration is provoked by spinal extension and rotation [26]. In the case of LBP related to SPL, pain worsens by prolonged static postures and/or movements within the so-called “neutral zone” according to Panjabi [27]. Difficulty falling asleep, waking up because of pain, pain worse with sitting and walking all demonstrated sensitivity > 0.75 for the presence of SPL in athletes [28].

When SPL is associated with compression of a nerve root in the lateral recess or in the foramen, patients may report paresthesia, reduction of sensitivity, and weakness in lower extremity [29]. In case of spinal stenosis, neurogenic claudication can be reported by patients together with difficulty in walking two to three blocks and doing their own shopping as well as getting in/out of a car [30–32].

A pain drawing completed by the patient is a simple tool for summarizing the characteristics of symptoms in a unique chart; however, it cannot identify the presence of psychological distress associated with LBP (e.g. anxiety, depression) [33]. The amount of pain can be reported using a Visual Analogue Scale or a Numerical Rating Scale [34].

The impact of SPL in terms of disability in activities of daily living (ADLs), including impact on sexual activity, can be assessed using the Oswestry Disability Index [35], which has demonstrated strong metric properties also in symptomatic lumbar SPL [36]. Other questionnaires useful for the assessment of cognitive-behavioural obstacles to recovery are: Fear Avoidance Beliefs Questionnaire and the Tampa Scale for fear of movement; the Coping Strategies Questionnaire and the Chronic Pain Coping Index for coping; the Pain Self-Efficacy Questionnaire for self-efficacy, the Pain Catastrophizing Scale, and the revised version of the Coping Strategies Questionnaire for catastrophizing [37]. The STarT Back tool can be administered to identify the risk of persistent lumbar disability [38]. A complete overview of the outcome measures properties is available in the COnsensus-based Standards for the selection of health status Measurement INstruments (COSMIN) checklist [39].

### Imaging

Static X-rays are the gold standard for the diagnosis of SPL when a translation > 3 mm in the sagittal plane is observed, and also considered as the threshold for “macroinstability” [40]. Standing lateral X-rays are more sensitive to identify degenerative SPL compared to conventional supine MRI [41, 42] . Furthermore, a discrepancy of spondylolisthesis grade measurements between weight-bearing X-ray and non-weight-bearing MRI has been demonstrated, suggesting a careful evaluation of both imaging techniques to determine the severity of SPL [43].

Dynamic flexion/extension X-rays are the gold standard for the diagnosis of unstable SPL, when a rotational movement > 10° or a translation > 3 mm in the sagittal plane compared to static X-rays are observed, a condition also defined as “microinstability” [44]. Although it is the most widely used method to diagnose abnormal vertebral motion, several concerns such as the best choice of patient position [45], the way that was used to analyse segmental mobility [46], and some errors in measuring translation in the sagittal plane [47] make its reliability and diagnostic value debatable.

Technologic advances in MRI (hard- and soft-ware), including vertical gap open MRI systems and functional MRI, allow investigation of spinal instabilities in a feasibly functional way with acceptable reproducibility [48].

In adolescent athletes with LBP, when it is important to identify spondylolytic pars stress fracture during early spondylolysis, the Single-Photon Emission Computed Tomography scan followed by lumbar Computed Tomography scan can identify the stress reaction process [49–51]. In young athletes CT scan is more accurate than MRI to diagnose spondylolysis [52].

### Physical exam

Clinical tests for symptomatic lumbar SPL can be divided into different types, depending on the aims of these tests, which include recognizing the presence of anatomical fault, assessing segmental mobility, provoking/alleviating pain and other symptoms as paraesthesia or dysesthesia, assessing motor control, and assessing lumbar muscles endurance [53, 54].

The most used test for recognizing the presence of forward slipping is the step-off sign/low midline sill sign, when the overlying spinous process is identified as anterior to the underlying one, during the inspection or palpation of lumbar spine in standing position. The low midline sill sign has shown sensitivity = 0.81, specificity = 0.89, positive predictive value = 0.78, and negative predictive value = 0.90 [55].

Concerning lumbar passive motion, the Posterior Shear Test [PST], also called the Segmental Spring Test or Passive Intervertebral Movement Test, aims to identify segmental hypermobility and/or provoke pain through passive posterior-anterior mobilization of the SPL level. This test demonstrated fair inter-examiner reliability, with k values from − 0.02 to 0.27 [56, 57]. Its specificity appeared generally high with values from 0.81 to 0.95 (positive likelihood ratios from 2.42 to 9.00), whereas its sensitivity was poor with values ranging from 0.17 to 0.46 (negative likelihood ratios from 0.60 to 0.88) [58].

Provocation/alleviation tests include the Prone Instability Test (PIT), the Passive Lumbar Extension Test (PLET), the Active Straight Leg Raising (ASLR), and the recently proposed Lumbar Rocking Test (LRT).

In the PIT the patient lies prone with the body on an examining table with legs over the edge and feet resting on the floor. While the patient rests in this position with the trunk muscles relaxed, the examiner applies posterior to anterior pressure to each vertebral segment of the lumbar spine. Any provocation of pain is reported. Then the patient lifts the legs off the floor (the patient may hold table to maintain position) and posterior to anterior compression is applied again to the lumbar spine while the trunk musculature is activated. The test is considered positive if pain is present in the resting position but subsides in the second position, suggesting lumbo-pelvic instability. Hicks and colleagues confirmed the strong diagnostic value of this test for establishing lumbar spine instability (sensitivity = 0.72; negative likelihood ratio = 0.48; specificity = 0.58; positive likelihood ratios = 1.7) [59].

The PLET test is performed in prone position; both lower extremities are passively elevated by the clinician to a height of about 30 cm from the bed while maintaining the knees extended and gently pulling the legs. This test is positive when it reproduces lumbar pain or feeling of instability and such symptoms disappear when the lower legs are repositioned to the starting position. The PLET test showed high sensitivity (0.70–0.93) and high specificity (0.82–0.95) in subjects with spinal stenosis or SPL or degenerative scoliosis [60] and a significant association with dynamic X-Rays (*P*-value = 0.017) in SPL [61]. A recent study confirmed its diagnostic value for establishing lumbar spine instability [62].

The ASLR is performed in supine position and the patient is instructed to lift the leg 20 cm off the bed by maintaining both knees extended. A positive response is pain or inability to lift the leg off the bed; however this response can vary from a slight difference in heaviness to complete inability. Next, an active or passive (using a belt) stabilization of the pelvis is applied to substitute or partially substitute the force required when the ASLR is painful or limited. A positive test is confirmed if pain/inability improves with stabilization [62, 63]. This test is separately scored on both sides as: 0 = not difficult at all; 1 = minimally difficult; 2 = somewhat difficult; 3 = fairly difficult; 4 = very difficult; 5 = unable to do. The scores of both sides are added, so that the summed score ranges from 0 to 10 [64].

The ASLR test demonstrated an interrater reliability ranging from 0.53 to 0.87 [65–67], sensitivity = 0.71 and specificity = 0.91 in females complained of lumbo-pelvic pain [67]. However, its accuracy in detecting lumbar instability in condition different from pelvic girdle pain is not known, and it did not appear related to pain or disability in SPL [61].

For the Lumbar Rocking Test, the patient lies comfortably in supine position on a table. The clinician induces a gentle jerk to the lumbar spine after locking hip and pelvis in hyper-flexed position by gently pushing knee onto the abdomen. If the subject complaints of severe pain in lumbar region while pushing the knee onto the abdomen, the test is considered to be positive. It has shown high sensitivity (0.95) and high positive predictive value (0.93) for lumbar instability [68].

The most commonly used motor control test for symptomatic lumbar SPL is the Aberrant Movements Test according to Hicks and colleagues [59] and Fritz and colleagues [57]. Painful arch in flexion, painful arch when returning from flexion, instability catch, Gower sign (lean with hands on thighs in flexion or back from flexion) and inversion of the lumbo-pelvic rhythm are the five components of this test. The relatively low sensitivity (from 0.18 to 0.26) and high specificity (from 0.72 to 0.88) suggest caution in the use of this test to diagnose lumbar instability [60].

Other specific tests aimed to assess the activity of deep stabilizers (transversus abdominis, multifidus, internal oblique, and so on) also can be performed in symptomatic lumbar SPL as in non-specific LBP. With respect to endurance, Bridge Tests (Supine Bridge Test, Prone Bridge Test, and Side Bridge Test) are the most used [69].

Overall, provocation/alleviation tests and endurance tests appear to be weakly related to the amount of pain but significantly related to disability in symptomatic SPL [61].

Among all these tests, the PLET exhibited the strongest relationship to positive dynamic radiographs [61, 62]. Bridge maneuvers showed to be responsive to detect clinical changes (pain and disability) after physical therapy treatment in symptomatic SPL [61, 69].

A clinical diagnostic rule for SPL has been proposed by Petersen and colleagues based on a cluster of tests including the step-off sign/low midline sill sign and the PST, associated with the PLET for degenerative SPL [26]. Neither PST nor PIT can be strongly recommended when used in isolation for testing lumbar instability [58].

At the end of the assessment, a clinician is able to perform a differential diagnosis among patients whose LBP is related to the presence of unstable SPL (in this case, we can expect positive instability tests), and patients whose pain may be related to different pain generators, when SPL is stable and instability tests are negative.

## Management and treatment

The presence of a lumbar SPL on imaging without relevant risks related to the slipping is not an indication for surgery, and conservative treatment is always preferable [70]. Despite the absence of consensus on the role of non-operative versus surgical care [71, 72], surgical indications are dependent by symptoms or other associated pathologic conditions rather than the severity/type of vertebral slippage [73]. Actually, taking into consideration the lack of association between LBP and lumbar spondylolysis (with or without SPL), surgical intervention for the adult general population in which spondylolisis/SPL provokes non-radicular LBP should be reconsidered [74]. According to a consensus conference on conservative treatment for degenerative lumbar spine stenosis (including SPL), a conservative approach based on at least 3 weeks of therapeutic exercise may be the first therapeutic choice in non-severe clinical conditions [75]. This same consensus conference concluded that physical therapy should use a multimodal approach and surgery should be considered if clinical condition does not change during 3 months or in presence of severe complication, e.g. lumbar radiculopathy or cauda equina syndrome [75].

Evidence supports the positive effect of the physical therapy on LBP due to spondylolysis and SPL [76, 77]. SPL-related pain without radiating pain may have the same characteristics as in non-specific LBP, in which classification into sub-groups, based on different clinical pictures, is considered critical to ensure appropriate management [19, 78, 79]. Some different LBP conditions are shown in the Fig. 1.

As suggested by Caneiro and colleagues [80], a person-centred active approach should be considered when treating musculoskeletal pain and disability including screening to identify biopsychosocial factors and health comorbidities and embracing patient-centred communication, educating using active learning approaches, and coaching towards self-management. Every patient complained of LBP, with or without SPL, can present different degrees of concurrent biological, psychological and social concerns. These unique factors must be considered to tailor objectives for each patient, both in the short- and long-term. Using StarT Back Tool as screening and adopting therapeutic tools such as the Modern Neuroscience Approach, Treatment-based Classification, and Cognitive Functional Therapy seem to predict a more favourable outcome with a specific treatment approach [81–84].

The aims of treatment will be different in isthmic and degenerative SPL. The typical patients with isthmic SPL are young, play sports, and active. In this case, the aim of physical therapy will be to restore the conditions for returning to previous activities with complete safety and without any fear of movement. This assumption requires that the recovered physical conditions (e.g. core stability, strength, endurance, coordination, etc.) should be better than before the onset of pain. These concepts also are relevant to degenerative SPL patients, but in this condition, the target goals could be less ambitious, albeit mandatory to restore the patient to painless ADLs.

### Self- management approach and graded supervised training

Systematic reviews [85, 86], clinical practice guidelines [87, 88] and international authors groups [89–91] indicate that self-management strategies are able to improve long-term outcomes in patients with chronic LBP. Self-management approaches should incorporate graded supervised training, during which the physical therapist incrementally increases the difficulty of the exercises, in line with the changes of the patient’s physical level and ability. However, limited evidence exists about the effectiveness of the graded exposure/graded activity for chronic [92] or persistent LBP [90]. In SPL patients, graded exposure/graded activity approach showed positive results only in case series [93, 94].

### The patient-therapist relationship and the importance of the first session

Within this framework, it is particularly important that the physical therapist induce positive expectations, starting from the first session. The setting, the therapeutic routine, the words used, the goals shared, the touch, and the initial manual therapy procedures may activate brain mechanisms having effects similar to a drug [95, 96]. Positive expectations also induce better treatment adherence; this is relevant for a therapy that lasts for months, given that the main results deriving from the exercises (e.g. less disability) may be shown only after some weeks of treatment

### Information and education

During all treatment, education must be a central component of patient care in order to facilitate behavioral changes. Taking into account the weak association between SPL and LBP, an SPL diagnosis must not create alarm for the patient [74, 97, 98]. Explanation of this weak association is extremely useful for improving patient compliance, which is essential for reaching clinically important outcomes [99]. Having received a message of diagnostic certainty from health practitioners, the patient can understand his/her pain in a more acceptable way. Patients who perceive diagnostic uncertainty, or receive a diagnosis of an underlying pathology that cannot be confirmed, are more confused and fearful [100]. Therefore, awareness of a clear diagnosis may counterbalance the negative influence of that diagnosis on pain self-efficacy and kinesiophobia [101].

Because poor self-efficacy and high fear of movements are associated with pain intensity and disability in SPL [25], health education and active strategies to enhance pain self-efficacy, decrease fear-avoidance beliefs and modify pain coping styles [21, 102–104] are also relevant in ishtmic SPL patients [93]. Education is an active process, dispelling unhelpful beliefs and building behavioral learning and self-efficacy regarding the safety and benefit of movement/activity. It is indicated in all patients with subacute or chronic LBP, whether it is related or not to SPL [105].

Relative to information and education, Ferrari and colleagues [25] tested the hypothesis that patients with SPL, who know they have a slipped vertebra, may exhibit less self-efficacy and more fear-avoidance behaviors compared to patients with non-specific LBP. The results of this study showed that this awareness did not significantly influence pain self-efficacy or kinesiophobia.

### Pain control

As higher perceived pain reduces pain self-efficacy and increases kinesiophobia [22, 24], it is mandatory to immediately use strategies to reduce pain. Besides the appropriate words use and correct information for activate biochemical and cellular brain mechanisms [95, 96, 106], the presence of different pain generators associated with the SPL suggest to recognize every individual pain source and treating it accordingly [107].

In addition, even if clinical guidelines found insufficient evidence to make a recommendation for or against the use of manipulation, some ancillary treatments such as traction, physical agents (e.g. TENS, superficial heat, low-level laser therapy) and other physical therapy procedures (e.g. massage, soft tissues treatment) could be used for limited time in the context of a multimodal, exercise-centred approach [31, 108]. Although the use of a brace is also debated [109–111], it can be used to facilitate ADLs with less pain, especially during the first weeks of treatment.

### Therapeutic exercise

Exercise can act on maladaptive primary (physical) and secondary (cognitive) compensations for movement and control impairments that promote ongoing pain. These subjects present with either an excess or deficit in spinal stability underlying their pain disorder.

Exercises should be consistent with the neurophysiology of motor control and should relate to the recovery of function. The proposed exercises must act on the patient’s motor knowledge (the so-called “internal model”), reactivating, improving, and reinforcing it. The internal model is useful not only to perform an action, but also to understand how that action should be carried out (slowly, quickly, coordinating it by regional interdependencies, etc.).

Demonstrating an exercise or action to the patient reactivates latent motor knowledge. In more complex cases, this concept can be followed using Action Observation Training or Mirror Therapy, consisting of an observation of actions performed by others, which activates the same neural structures responsible for the actual execution of those same actions in the perceiver [112, 113].

**Fig. 2:**
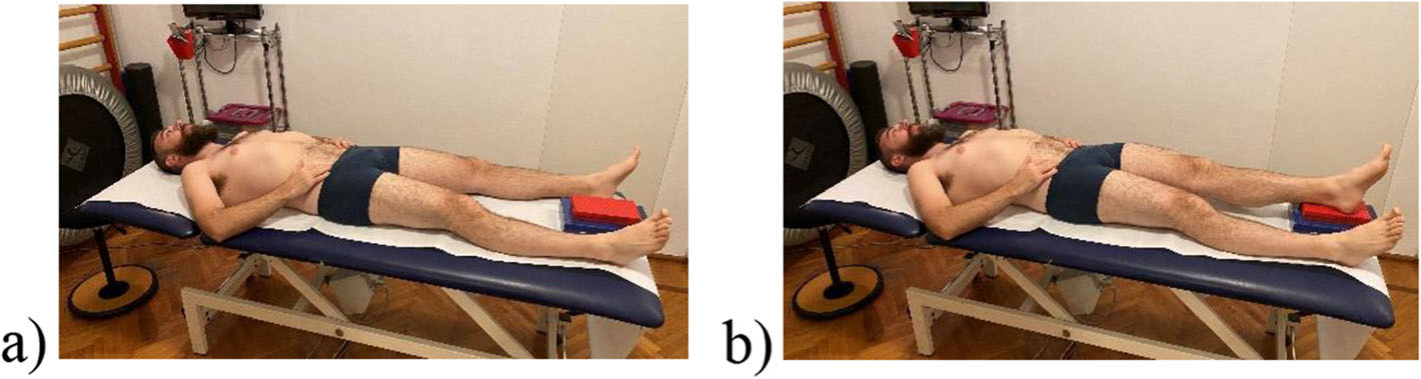
Example of exercise for subjects with positive Active Straight Leg Raising. The subject slowly raises his/her legs, alternatively, on a little box laid between the feet, and go back. Using his/her fingers, the subject checks to not move or turn the pelvis, enhancing motor control at the starting, during the movement, and at the return on the starting position. This exercise may be performed ten to twelve times for each leg, every day

**Fig. 3:**
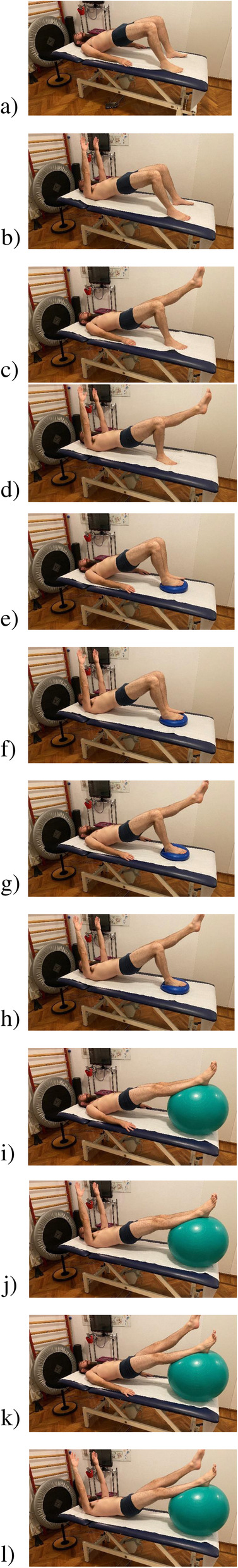
Example of progressions for Supine Bridge exercise, which can be implemented step by step, based on the ability of the patient. For example, Supine Bridge exercise performed with both feet on the ground should be maintained for 60 s and repeated three times. Same exercise performed with only one foot on the ground should be held for less time, with high repetitions

Every exercise must address a specific goal. For example, it has been suggested using same clinical tests that initially were performed with difficulty as exercises. If the Active Straight Leg Raise was positive, the exercise showed in Fig. 2 may be proposed; if the Supine Bridge Test was performed with difficulty and/or maintained for only a short time, the same Bridge exercise may be proposed. Supine Bridge exercise can be taken as an example of graduating exercises because it allows an incremental increase of difficulty by adding some new and challenging variations (see Fig. 3).

**Fig. 4:**
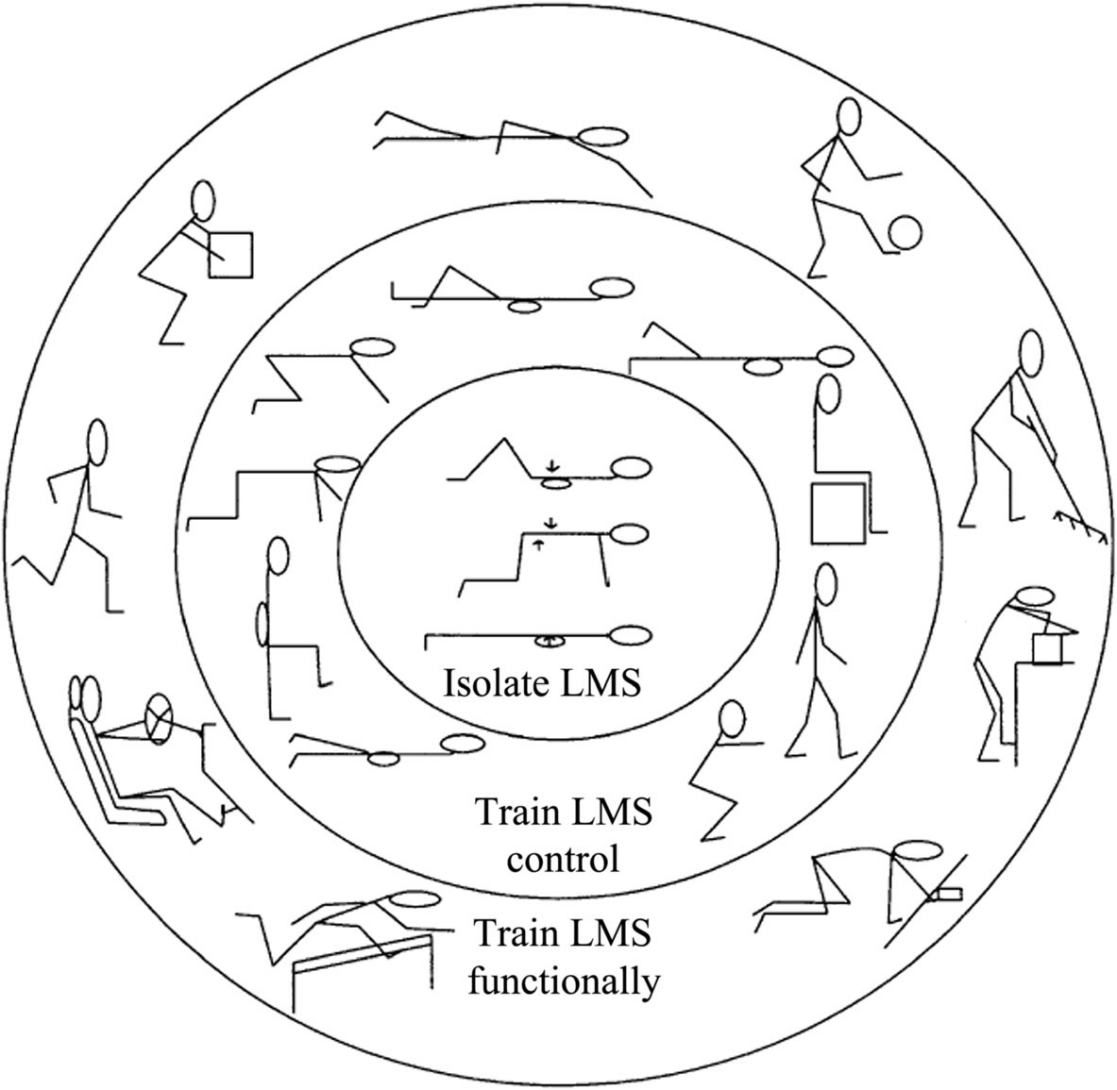
The model of motor control training, according to O′ Sullivan (from Twomey L. T. & Taylor J.R. Physical Therapy of the Low Back, 3rd edition, 2000). Reproduced by kind permission of W.B. Saunders

**Fig. 5:**
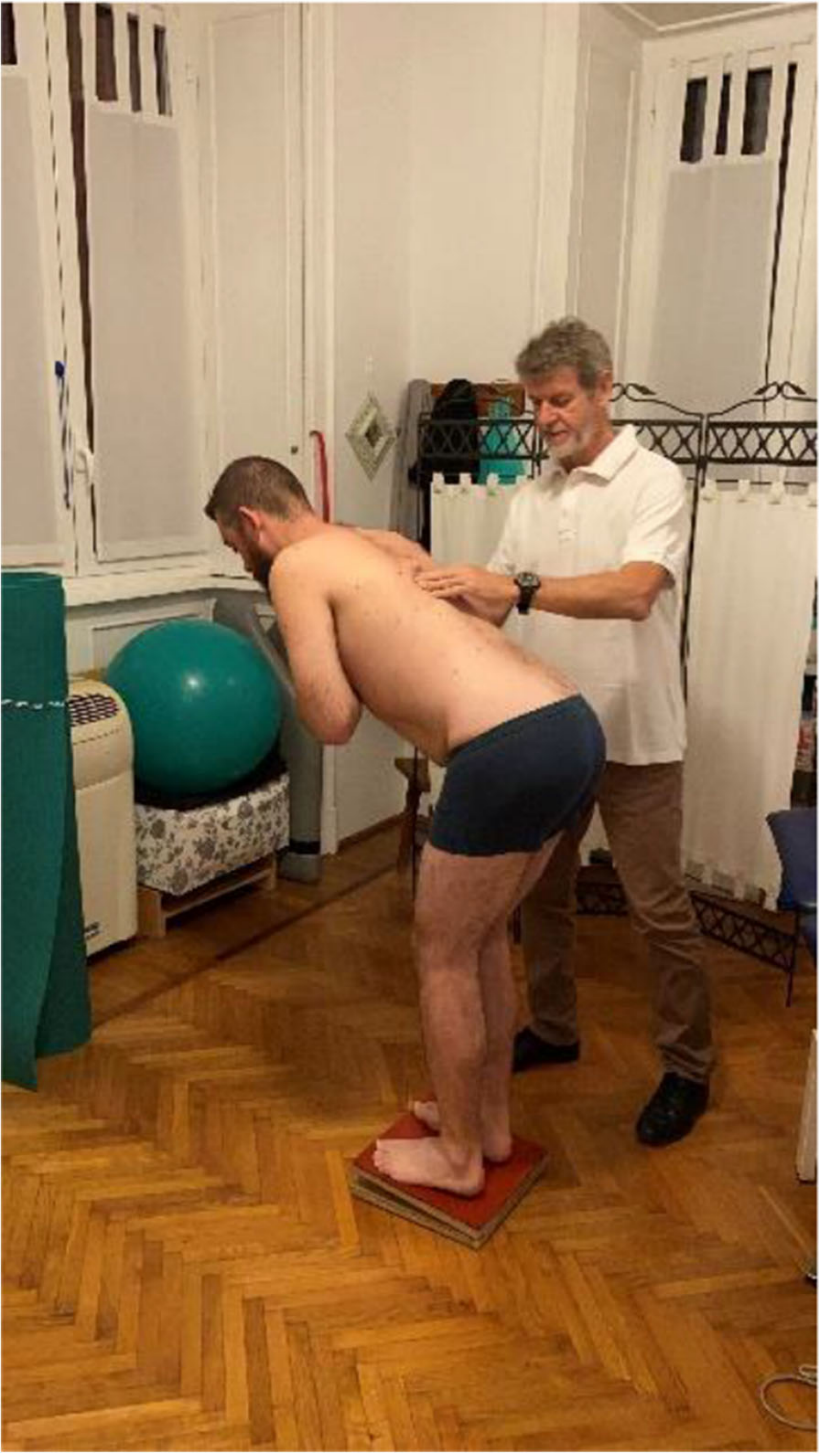
Example of exercise for lumbar multifidus muscle using a Global Postural Reeducation posture. The subject has to maintain the lumbar spine in a stable and neutral position while slowly bends forward the trunk up to 45 degrees, avoiding any giving towards kyphosis or hyper-lordosis. This slow bending forward exercise should last for at least 30 s and should be repeated 3 to 5 times

The model previously proposed by O’Sullivan for motor control training is still current [109, 114–116], addressing the activation of stabilizer muscles at rest and during contraction, together with reducing the overuse of superficial muscles [7, 8] (see Fig. 4). Among local stabilizers, paying attention to the recovery of the lumbar multifidus seems mandatory. Although the review by Pillastrini and colleagues [117] was not able to identify which exercise best modifies the multifidus structure, it showed that its thickness and/or cross-sectional area may increase when more than one exercise is performed, progressing from motor control to increasing static and dynamic loads. An exercise for the multifidus muscle using a Global Postural Reeducation posture is shown in Fig. 5 [118]. The core of training must be performed in lying, seating and standing positions. After some practice sessions, as the patient’s abilities permit, the same initial exercises should be performed in unstable conditions, e.g. using devices like Swiss balls or proprioceptive devices. At the end, as muscle imbalance has been associated with bad posture and functional disability [31, 119], the aim of the physical therapy program should be achieving trunk muscle balance rather than muscle strength alone.

Many complaints from SPL patients concern the capacity of walking and standing up for long time. For this reason, endurance exercises must be proposed. Since the stabilizing activity of core muscles is generally characterized by a low-intensity contraction [120], common exercise protocols focus on high repetitions and low-load contractions. At about the middle of the entire treatment, a specific walking-based training should be initiated, starting with slow-velocity walking and progressing toward faster walking. For younger, active patients this walking program can be progressed up to running. Of course, this goal is mandatory in subjects who engage in sports.

A model of a whole physical therapy program in typical SPL patients has been proposed by Ferrari and colleagues [93] (see Table 1).

**Table 1:** Model of physical therapy program in symptomatic lumbar SPL, proposed by Ferrari and colleagues (from: Ferrari S, Vanti C, Costa F, Fornari M. Can physical therapy centred on cognitive and behavioural principles improve pain self-efficacy in symptomatic lumbar isthmic spondylolisthesis? A case series. J Bodyw Mov Ther. 2016;20 [3]:554–64). Reproduced by kind permission of … . (editor)

	Education	Ergonomics	Supervised Exercises	Home exercises
Session #1	Spondylolisthesis: origin and transmission of lumbar pain	The sitting posture: at home, at work, driving car, with or without lumbar supports	Awareness of sitting and standing positions Lumbar muscles contraction and relaxation	Exercises in lying position: bring one or both knees to the chest Supine bridge exercise slowly
Session #2	Modulation of pain: descending pathways, role of drugs and exercise	The standing posture: at home, at work, etc.	Isometric activation of local stabilizers in supine, quadrupedal, sitting and standing positions Maintaining muscle contraction for 10 s and breathing normally Dynamic tasks, from the easiest to the most difficult, maintaining muscle stabilization and five seconds of static contraction between movements.	Active exercises in standing position Dynamic simple upper and lower limb movements
Session #3	Active and passive coping strategies: different effects	Load carrying, with or without lumbar support	Start of the progressive supervised physical and functional graded activity Start of functional recovery of balance and coordination	Start of Swiss ball exercises Start of aerobic activity (i.e., walking, cycling, swimming)
Session #4	Previous lessons review, to mark the right procedure: pain ➔ awareness ➔ active coping ➔ physical activity	Practical simulation of posture and load management	Starting of functional recovery of strength, endurance, and range of motion	Progression of exercises in lying, standing, sitting position Progression of aerobic activity
Sessions from #5 to #10	Prognosis of lumbar Spondylolisthesis	Repetition and reinforcement of concepts	Progression of functional recovery of strength, endurance, range of motion, balance and coordination	High-loading exercises Nordic walking, dance or Pilates procedures

#### Passive mobilization

A useful adjunct treatment may be the mobilization of stiff spinal segments. Mohanty & Pattnaik [17] proposed mobilization of thoracic spine as an adjunct to stretching legs and core stability exercises, based on the concept that decreasing thoracic hypomobility should also decrease the hypermobility of painful lower segments. Some controversies exist about manual therapy, often used in the context of a multimodal treatment, regarding the indication for repeated end-range extension movements [71, 82].

#### SPL with radiating pain and/or lumbar stenosis

In this case, conservative therapy comprising physical therapy, epidural steroid injection, and pain medications may be considered as first step [75, 121]. If unresolved, surgical options may include decompression alone or decompression and fusion [122].

With respect to physical therapy, a recent consensus publication by experts suggested a multimodal approach (exercises, manual therapy, information and education) for symptomatic lumbar stenosis, even when caused by SPL [75]. However, there is insufficient evidence to make a recommendation for the use of other physical therapy interventions such as aquatic therapy, acupuncture, psychosocial intervention, transcutaneous tibial nerve stimulation, and neural mobilization [75].

As multifidus atrophy has been also found in patients with lumbar radiculopathy [123], these subjects need the same motor control exercises mentioned above. Positive effects in lumbar stenosis have been obtained by spinal and lower limb muscle stretching, spine and pelvis mobilization, strenght training, walking on treadmill and stationary cycling [124].

Although the effectiveness of TENS, belts and traction is debated [125–128], these treatments could be used in specific patients within the context of a multimodal program to improve pain and allow the performance of specific exercises. Moreover, although there is no evidence concerning neural mobilization treatment beyond the anecdotal, this specific approach could be proposed to reduce neural mechanical sensitivity [129].

#### SPL complicated by other factors

A challenging group of SPL patients are those patients showing features suggestive of central sensitization, a process characterized by generalized hypersensitivity of the somatosensory system [130, 131]. Similar to individuals with chronic spinal pain [132–134], SPL patients also may need pain neuroscience education combined with cognition-targeted motor control training for improving symptoms, mental and physical functioning, enhancing pain cognitions, and reducing disability.

A different treatment goal applies to adult/older age subjects with SPL associated with severe disk degeneration. In these cases, the focus of physical therapy should be on a specific stabilizing program aimed at reducing pain and disability while waiting for a possible spontaneous stabilization. This outcome was demonstrated on a 44 year old woman with a grade II SPL after six-years treatment [94].

#### SPL in adolescents

In general, there is no justification for generally advising children and adolescents with isthmic SPL to limit or avoid competitive sports [135, 136]. On the contrary, with proper treatment, excellent outcomes occur [2, 4].

In the acute phase, e.g. after a trauma, an early diagnosis can lead to healing of the pars interarticularis defect after stopping sports activity and a period of brace immobilization [for 6–8 weeks or more] [5, 137]. Also low-intensity pulsed ultrasound, in addition to conservative treatment, has been suggested for achieving a higher rate of bony union [138–140].

#### Treatment timing and dosage

Type, dosage and progression of exercises are related to both SPL and subject characteristics. For example, they can vary between isthmic and degenerative SPL, because age, basic physical activity and objectives may be different. In any case, research indicates that treatment should overcome the patient’s limits, using challenging exercises capable of improving the subjective physical performance, without psychological hesitation. One weekly session initially (for a total of 4–5 sessions), followed from one session every 15 days, then monthly sessions, may be useful to achieve treatment needs in each different patient, whether it is a sportsman, a housewife, or a farm worker. The proposed dosage is frequent enough to manage impairments and long enough to achieve physical, cognitive and behavioral changes. The frequency of sessions can increase in case of severe pain by combining manual therapy or physical therapy procedures.

How long should the treatment of symptomatic lumbar SPL last in adults and older adults? O’Sullivan’s study specified that the intervention period corresponded to 10 weeks [115], whereas Ferrari and colleagues in their case series reported that session number and total treatment time largely varied with a range from 4 to 10 sessions lasting 2–4 months [93]. A retrospective cohort study of consecutive adult patients admitted for physical therapy with symptomatic lumbar grade I SPL found that the number of sessions required to achieve satisfactory outcomes ranged from 5 to 12. Interestingly, the clinical outcomes achieved in the 5–8 sessions group were similar to the 9–12 sessions group [141], suggesting that fewer sessions can obtain positive results.

## Conclusions

The management of symptomatic lumbar SPL should consider the type of SPL, the presence or absence of instability and neurological symptoms, the stage of pain, and the cognitive-behavioral framework. An accurate assessment is essential to define the characteristics of each individual patient and design a tailored treatment program based on main lumbar dysfunctions. However, taking into consideration that the current literature has been focused on metric properties of individual tests; we do have not enough data to suggest the best cluster of clinical tests to be used.

An integrated treatment plan, including pain management, education, supervised exercise, self-treatment, and physical activity is essential to enhance the patient’s ability to meet the challenges of this condition. Outcome measures should concern not only pain, but also spinal function, general and local fitness, daily activities, and psychological aspects such as fear-avoidance, pain catastrophizing, and pain self-efficacy. The best result would be to teach patients to manage their own specific conditions.

Defining different symptomatic lumbar SPL subgroups and investigating the effects of treatments based on that classification, similar to the approach already proposed for non-specific LBP, are some suggestions to improve the diagnostic-therapeutic approach in SPL.

## Data Availability

We agree to the terms of the BioMed Open Data policy. This article did not require numerical data nor statistics.
